# Modern Surgery of the Stomach1Read before the Northwestern Medical Society of Philadelphia, April 5, 1909.

**Published:** 1909-09

**Authors:** John B. Deaver

**Affiliations:** Philadelphia, Pa.


### Modern Surgery of the Stomach[1]

BY JOHN B. DEAVER, M. D., Philadelphia, Pa.

(*Richmond Journal of Practice*, July, 1909.)

TODAY the chief problems of surgery lie in a region which may be covered by the palm of the hand placed on the epigastrium.

Surgery of the appendix, Fallopian tubes and biliary apparatus has already been elucidated. The early operations for gastric ulcer and cancer have been amplified and improved, because it has been shown that in these alterations of the stomach expectant methods, assisted by drugs and hygiene, fail to work a

1. Read before the Northwestern Medical Society of Philadelphia, April 5, 1909.

cure. Of all diseases of the stomach, ulcer is the most important, it is not so uniformly deadly as cancer, but it is vastly more common, and, beside, a large percentage of these cases result in malignant change. Including in this category the duodenal ulcers, it may be said that one out of every twenty individuals has been at some time the subject of this affection. It is therefore evident that the disease either gives no symptoms in many cases, or that they fail in recognition. It is repugnant to our ideas of an ulcerated process to suppose that it can exist without symptoms, and, in the presence of such a multitude of cases of indigestion, it would seem to be going rather far afield to ascribe these symptoms to undiscoverable causes and leave ulcer the symptomless wonder of the clinician. It is plain that the symptomatology of this condition must be enlarged, that we must not make diagnosis dependant upon such conditions as pain of a certain type, with nausea and vomiting, hematemesis and melena. Inability to carve out a sufficiently definite symptom-complex to render possible an early diagnosis should not lead to discouragement, but should rather be an incentive to greater effort.

Ulcer is vastly more frequent than our diagnoses would indicate, and we must, therefore, be treating many cases in a haphazard manner for dyspepsia who are really suffering from ulcer, thus permitting a certain percentage to go on to perforation, hemorrhage or the callous stage.

Statistics show that recurrence takes place in nearly half the cases after "medical cures." The mortality of recognisable cases of gastric ulcer ranges from 10 to 50 per cent., while in duodenal ulcers it is somewhat higher. It is plain, therefore, that in diagnosable ulcer one should not temporise. The indication is rest for the part, and not only rest, but a long rest. This factor removes most of these cases from the sphere of the medical man to that of the surgeon, as it is impossible to provide rest by medical means sufficiently long to permit a cure in the majority of cases. The efficiency of gastric drainage has been amply demonstrated, and eminent surgeons are a unit as regards the remarkable results obtained in this way. The mortality of gastro-enterostomy in the best hands is only from 1 to 3 per cent., and is constantly diminishing. The results of seven eminent surgeons in nearly a thousand cases show 73 to 93 per cent. of cures, which is wonderful, considering that only the most severe cases have been turned over to the surgeon. Gastroenterostomy, therefore, is the treatment of choice in simple, chronic, persistent, symptom-giving ulcers of the stomach and duodenum.

The complications of ulcer are various, and include perforation, which may be acute or chronic, and lead to peritonitis, extensive adhesions, subphrenic or other forms of abscess; hemorrhage, which may be slight, or even so severe as to be fatal from erosion of the coronary artery—conditions which call for immediate operation. Spontaneous cure brings with it special dangers. Cicatrisation of an ulcer at the pylorus is a not infrequent cause of stenosis, with subsequent gastric dilatation; in this latter condition tetany occasionally develops. These conditions are readily relieved by gastro-enteroanastomosis.

In dealing with uncomplicated ulcer, where it is desirable simply to secure drainage, gastroenterostomy, by the posterior no-loop, or very short method, uniting the most dependent portions of the stomach with the jejunum at its origin behind the peritoneum, has proved the best in my hands. I have not seen a case of vicious circle follow one of these operations. In regard to the direction of the incision in the stomach, I have usually inclined it slightly to the right from above downward, suturing the proximal portion of the jejunum to the upper angle, but have lately made the opening incline to the patient's left with equally good results. In cases of simple stenosis of the pylorus, without dilatation, a pyloroplasty, after the method of Finney, is to be preferred, but gastro-jejunostomy gives as good a functional result. If the stomach wall is greatly distended or hypertrophied, the latter operation is better. Caution in diet after operation is advocated, because neglect of this defeats the object of procuring rest for the diseased area.

Nowhere do we see the need of a professor of practical medicine in our schools more than in connection with cancer. Cachexia, hematemesis, mass, enlarged liver and anacidity are symptoms not of cancer, but of approaching dissolution. The reason why carcinoma of the stomach is relatively so much more deadly than carcinoma of the lip or breast is simply that an early diagnosis by direct inspection and palpation cannot be made in cancer of the stomach without the formality of an operation. There is little use of a practitioner bothering his head about a diagnosis if he insists on certainty. By the time he is certain of his diagnosis he is also certain of the prognosis. Therefore I am inclined to say that a positive diagnosis is a disgrace to the physician in attendance, providing he has been following the case for some weeks or months. The latest device which has been invented to defer operation until it is too late, is gastric analysis. I have had for some years routine examination of the stomach contents made in all cases of upper abdominal disease in which the stomach may be affected, including disease of the gall-bladder, but disappointment has been so frequent and great that I have come to place no reliance on the method. The last fifty examinations subsequently controlled by operations show that anacidity is not infrequent in diseases other than that of the stomach; that in early carcinoma the acidity is but slightly altered; but in late inoperable carcinoma a but slightly subnormal acidity may occur; that anacidity has almost invariably gone hand in hand with an extensive inoperable growth; blood indicates ulceration, which does not occur in the early stages; that the Oppler-Boas bacillus occurs only in the late stages of cancer, and may be found in conditions of stasis from benign causes. The *x*-ray gives no aid of any moment. Where surgery will do the most the *x*-ray will do the least.

The patient approaching or past middle life, who, either suddenly or on top of previous gastric disturbances, finds that his digestion is rapidly getting worse; has little inclination to eat some articles of food, often meat; feels a certain languor and lack of vigor; has eructations of gas, regurgitation, a slight feeling of discomfort in the epigastrium or left hypochondrium, and begins to lose weight in the absence of a very definite cause outside of the stomach, should have his stomach inspected directly. This will at times be needless, but in a large percentage of these cases we will find early neoplasm curable by extirpation

The ideal treatment for cancer is complete excision. In the majority of cases it is located in the pyloric region, and, by a fortunate arrangement of the lymphatics, does not tend to spread to the fundus, but the glands of lesser curvature and the adjacent gastro-hepatic and gastro-colic omentum are early involved. A wide excision, embracing these structures, gives hope of circumventing the disease in the early stages. My method is essentially the Billroth No. 2, which consists in removal of that part of the stomach lying between the first portion of the duodenum and the Mickulicz-Hartmann line, which is an imaginary perpendicular drop from the cardiac orifice to the greater curvature. The cut ends of the stomach and duodenum are closed, and a posterior gastro-enterostomy done in the usual manner. A complete gastrectomy is rarely indicated. The surgical treatment of advanced carcinoma, in which it is hopeless to get beyond the growth, is a very unsatisfactory part of surgery. The most frequent call for palliative operations comes from pyloric stenosis, with retention. In such cases gastroenterostomy often gives marked temporary relief, and adds about three months to the expectation of life. In obstruction of the cardiac orifice, death from starvation may be prevented by gastrostomy, which is, however, not indicated so long as the patient can swallow liquid. In the future I hope there will be fewer explorations in these cases with a positive diagnosis, to see whether something can be done—a hope practically always illusory and more explorations in suspicious cases while there is yet a chance of cure.

It is necessary to have constantly in mind those minor and functional disorders of the stomach, in which ill-advised operations can hardly be expected to result otherwise than in failure. Such cases may be hard to differentiate, but they usually show other neurotic stigmata, and are very susceptible to any change in treatment or hopeful suggestion.

Gastroptosis is usually associated with general visceroptosis, and an operation on any one of the displaced viscera promises little. I am not inclined to recommend operation in cases of ptosis, and certainly not until the effect of a properly-fitting abdominal supporter and general medical treatment is tried. In gastrectosia, in the presence of a patulous pylorus, operation may be serviceable, and give truly brilliant results, but in such cases medical treatment should be given a thorough trial before operation is done. The best operation is posterior gastroenterostomy, done at the most dependent part of the stomach. I have never seen a case of acute dilatation of the stomach, and have come to the conclusion that the reason for this is the early and free use of the stomach-tube in all cases that show accumulation of fluid or gas in the stomach.

The chief point to emphasize, however, in all this subject, is that we must not allow the frequency of minor and functional disorders to divert our attention from the common occurrence and serious nature of ulcer and cancer. Ten years ago gastric surgery was far behind diagnosis. Today it is just as far ahead, and we wait, perhaps impatiently, for the internist to come abreast.

1634 Walnut Street.

